# Sleepiness and Vitamin D Levels in Patients with Obstructive Sleep Apnea

**DOI:** 10.3390/healthcare12060698

**Published:** 2024-03-21

**Authors:** Kostas Archontogeorgis, Nicholas-Tiberio Economou, Panagiotis Bargiotas, Evangelia Nena, Athanasios Voulgaris, Konstantina Chadia, Georgia Trakada, Andrea Romigi, Paschalis Steiropoulos

**Affiliations:** 1 Department of Pneumonology, Medical School, Democritus University of Thrace, 68100 Alexandroupolis, Greece; k.archontogeorgis@yahoo.it (K.A.); nt_economou@yahoo.it (N.-T.E.); thanasisvoul@hotmail.com (A.V.); con_chadia@yahoo.gr (K.C.); 2 Sleep-Wake Lab, Department of Neurology, Medical School, University of Cyprus, Nicosia 2408, Cyprus; bargiotas.panagiotis@ucy.ac.cy; 3 Laboratory of Hygiene and Environmental Protection, Medical School, Democritus University of Thrace, 68100 Alexandroupolis, Greece; evangelianena@yahoo.gr; 4 Division of Pulmonology, Department of Clinical Therapeutics, School of Medicine, National and Kapodistrian University of Athens, Alexandra Hospital, 11635 Athens, Greece; gtrakada@hotmail.com; 5 IRCCS Neuromed Istituto Neurologico Mediterraneo Pozzilli (IS), 86077 Pozzilli, Italy; andrea.romigi@gmail.com; 6 Faculty of Psychology, Uninettuno Telematic International University, 00186 Rome, Italy

**Keywords:** vitamin D, sleepiness, obstructive sleep apnea (OSA), sleep apnea, Epworth sleepiness scale (ESS)

## Abstract

Study Objectives: The aim of this cross-sectional study is to explore the association between serum 25-hydroxyvitamin D [25(OH)D] levels, a marker of Vitamin D status, and excessive daytime sleepiness (EDS), expressed as increased scores of the Epworth Sleepiness Scale (ESS), in a group of prospectively enrolled patients with obstructive sleep apnea (OSA). Methods: Newly diagnosed patients with OSA, divided into two groups, those with EDS (ESS > 10) and those without EDS (ESS < 10). All patients underwent night polysomnography. Measurement of serum 25(OH)D vitamin was performed using a radioimmunoassay. Results: In total, 217 patients with OSA (197 males and 20 females) were included. Patients with EDS had higher AHI (*p* < 0.001) values and lower mean serum 25(OH)D levels, compared with those of non-somnolent patients [17.4 (12.2–25.7) versus 21.1 (15.3–28.8) ng/mL, respectively, *p* = 0.005]. In patients with EDS, serum 25(OH)D levels correlated with average oxyhemoglobin saturation during sleep (r = 0.194, *p* = 0.043), and negatively with ESS score (r = −0.285, *p* = 0.003), AHΙ (r = −0.197, *p* = 0.040) and arousal index (r = −0.256, *p* = 0.019). Binary regression analysis identified Vit D serum levels (β = −0.045, OR: 0.956, 95% CI: 0.916–0.997, *p* = 0.035), total sleep time (β = 0.011, OR: 1.011, 95% CI: 1.002–1.021, *p* = 0.016) and AHI (β = 0.022, OR: 1.022, 95% CI: 1.003–1.043, *p* = 0.026) as independent predictors of EDS in patients with OSA. In patients with EDS, multiple regression analysis indicated that ESS score was negatively associated with Vit D serum levels (β = −0.135, *p* = 0.014) and minimum oxyhemoglobin saturation during sleep (β = −0.137, *p* = 0.043). Conclusions: In the present study, EDS in patients with OSA is associated with low levels of Vitamin D, while sleep hypoxia may play a role in this process.

## 1. Introduction

Obstructive sleep apnea (OSA) is characterized by intermittent cessation of breathing during sleep due to partial or complete upper airway obstruction [1]. By definition, these episodes are always accompanied by respiratory effort and result in oxyhemoglobin desaturation and sleep fragmentation [1]. Currently, OSA is the most frequent sleep-related breathing disorder, with an estimated prevalence between 10–17% for men and 3–9% for women in the Western countries [2]. OSA-related symptoms could be divided into nocturnal, such as loud snoring and a choking sensation during sleep, and diurnal, such as excessive daytime sleepiness (EDS), which among others is recognized as the most common and disabling daytime feature of OSA [3].

EDS is a frequently reported symptom of OSA and it may affect from 19% to 87.2% of patients diagnosed with OSA [4]. Although the prevalence of EDS in OSA exhibits significant variability in epidemiological studies, these evident differences in the prevalence data might be attributed to a complex interplay of several factors, including the methodologies employed for EDS measurement, sample sizes, age, gender, severity of OSA, ethnicity, the presence of comorbidities, and other unrecognized factors that could impact OSA manifestation [5]. EDS is characterized by the inability to remain alert and maintain wakefulness during the day, with sleep occurring unintentionally or at inappropriate times almost daily for at least three months [6]. Well-studied risk factors for this condition include increased BMI and neck circumference, older age, male gender and anatomical variations causing narrowing of the upper airway [7]. As the syndrome evolves, and while at the same time remains undiagnosed and untreated, EDS becomes debilitating resulting in impaired quality of life, reduced work performance and increased probability for traffic accidents [3]. 

Information regarding Vit D is now widely accessible not only in the scientific literature, but also across various platforms on the internet [8,9]. Vitamin D (Vit D) is a fat-soluble vitamin produced in the skin after exposure to solar radiation. Vit D exists in various forms and serum 25-hydroxyvitamin D [25(OH)D] is considered the marker of choice for the measurement of Vit D levels [8]. Interestingly, Vit D insufficiency (defined as Vit D levels < 20 ng/mL) and deficiency (defined as Vit D levels < 10 ng/mL) has an increased prevalence worldwide and is present in numerous pulmonary diseases, such as viral and bacterial respiratory infections, asthma, chronic obstructive pulmonary disease and cancer [10]. Recent data reported lower Vit D serum levels in patients with OSA compared with non-apneic individuals, while CPAP treatment showed beneficial effects on Vit D concentrations in this particular population of patients [11,12]. 

Data regarding the relationship between Vit D serum levels and EDS in OSA are still scarce. Hence, the aim of the present cross-sectional study is to compare Vit D serum levels in OSA patients with and without EDS and to explore potential associations between Vit D levels and the degree of excessive daytime sleepiness in these patients. In addition, secondary endpoints of the study are to examine possible correlations between Vit D levels and different anthropometric and sleep characteristics, focusing on the subgroup of patients with EDS.

## 2. Materials and Methods

### 2.1. Patients

Patients who underwent polysomnography in our Institution and were consecutively diagnosed with OSA were included in the study. 

Patient recruitment took place between 1 April and 31 October 2017, in order to avoid significant variations in exposure to sunlight, which could affect Vit D levels. The following exclusion criteria were applied: Vit D supplementation, central sleep apnea syndrome, corticosteroid and/or diuretic therapy, conditions known to affect calcium, phosphorus and Vit D metabolism and absorption, heart failure, inflammatory diseases, cancer, liver or kidney disease, osteoporosis and patients with no OSA-related EDS [13].

Overall, 289 patients with OSA [250 males and 39 females with a median age 57 (48–65) years] were evaluated for inclusion in the present study. A detailed medical history regarding past medical conditions, known comorbidities, current medication use, focusing on Vit D supplements, and tobacco smoking was recorded. A clinical examination and the assessment of anthropometric characteristics (including height, weight, neck circumference, hip, and waist circumference as well as waist/hip circumference ratio and body mass index-BMI) were performed.

OSA was defined as AHI ≥ 15 events/hour of sleep or as AHI ≥ 5 events/hour of sleep accompanied by symptoms of disturbed sleep, such as excessive daytime sleepiness, gasping or choking during sleep, observed loud snoring or breathing interruption [3].

Sleepiness was evaluated using the validated Greek version of the Epworth Sleepiness Scale (ESS) [14], a self-administered questionnaire which includes eight questions referring to typical everyday situations. By attributing a score from 0 to 3, the patient rates the possibility of falling asleep in each situation. A score > 10 indicates EDS.

Pulmonary function testing, analysis of arterial blood gases and a 12-lead electrocardiogram were also performed in order for potential coexistent pulmonary and cardiovascular diseases to be excluded.

### 2.2. Polysomnography

An attended overnight polysomnography was performed from 22:00 to 06:00 h and variables were recorded on a computer system (Alice^®^ 4, Philips Respironics, Murrysville, PA, USA). A standard montage of electroencephalogram, electro-occulogram, electromyogram and electrocardiogram signals was used for sleep staging. Pulse oximetry was registered and airflow was detected using combined oronasal thermistors for apneas and nasal pressure for hypopneas. Chest and abdominal motion were detected using inductive plethysmography. Sleep staging and respiratory events were scored manually according to standard criteria [15]. Apnea was defined as a ≥90% of reduction in airflow for at least 10 s [15]. Hypopnea was defined as a ≥30% reduction in airflow for at least 10 s in combination with oxyhemoglobin desaturation of at least 3% or an arousal registered by the electroencephalogram [15]. The apnea–hypopnea index (AHI) was calculated as the average number of apneas and hypopneas per hour of PSG-recorded sleep time [15]. 

### 2.3. Blood Samples and Measurements

Venous blood samples were collected from all participants the morning after polysomnography after at least 8 h of fasting. Samples were obtained from the antecubital vein and were left to coagulate and then centrifuged (3000 rpm for 10 min). Biochemical parameters regarding renal and liver function, as well as glucose, C-reactive protein (CRP) serum levels and lipid profile were measured using an automated analyzer. Serum concentrations of 25(OH)D were determined using a commercial radioimmunoassay kit and the manufacturer’s specifications the same day as that of the blood sampling (DiaSorin, Stillwater, MN, USA). 

### 2.4. Statistical Analysis

The sample size was determined by using the G*Power software version 3.1.9.7. It was evaluated that the minimum sample size to yield a statistical power of at least 0.8 with an alpha of 0.05 and a medium effect size (d = 0.35) was 102. All analyses were carried out using IBM Statistical Package for Social Sciences (SPSS Inc. Released 2008. SPSS Statistics for Windows, Version 17.0. Chicago, IL, USA: SPSS Inc.). The normality of distribution for continuous variables was tested with the Shapiro–Wilk test. All data are expressed as the median (25th–75th percentile). Comparison of percentages between groups was performed with the chi-squared test. In normally distributed variables, correlations were analyzed with Pearson’s correlation coefficient, while comparisons between means were studied with the Student’s *t*-test. In case of skewed distribution, the Spearman’s correlation and non-parametric Mann–Whitney test was applied. Independent predictors of EDS between the two groups were identified using binary logistic regression analysis. In the EDS group, independent factors of EDS were further determined using multiple linear regression analysis. Reported *p*-values are two-tailed and significance was defined at *p* < 0.05. 

### 2.5. Ethics Approval

All procedures were carried out in accordance with the Helsinki Declaration of Human Rights and patients gave their informed consent [16]. The study protocol was approved by the Institutional ethics committee of the University General Hospital of Alexandroupolis (approval date: 13 October 2014).

## 3. Results

In total, 217 patients with OSA (197 males and 20 females) participated in the present study. Included patients were middle-aged [age was 55 (46.5–62.5) years] and obese [BMI was 35.3 (31.4–38.3) kg/m^2^], while median serum Vit D levels were of 19.4 (13.3–26.5) ng/mL. Females exhibited lower 25(OH)D serum levels compared with males [15.5 (9.7–20.6) versus 20.2 (13.4–26.7), respectively, *p* = 0.048)]. 

Participants were divided according to the presence or not of EDS into two groups: non-sleepy (ESS score ≤ 10), which included 108 patients (96 males and 12 females), and sleepy (ESS score > 10), which included 109 patients (101 males and 8 females). No differences were identified between the two groups in terms of gender, age, and BMI. Anthropometric and demographic characteristics of included patients are presented in Table 1.

In sleepy patients, the following findings were demonstrated: a longer total sleep time, higher sleep efficiency, higher values of arousal index, AHI and worse indices of hypoxia during sleep when compared with patients without EDS. Sleep characteristics of patients are presented in Table 2.

Additionally, patients with EDS had a poorer lipidemic profile, as expressed by higher triglycerides levels and lower HDL-C, compared with patients without EDS. Moreover, sleepy patients with OSA had significantly lower serum 25(OH)D levels than those without EDS [21.1 (15.3–28.8) versus 17.4 (12.2–25.7) ng/mL, respectively; *p* = 0.005]. Results of blood examinations of patients are presented in Table 3 and results from pulmonary function testing are presented in Table 4.

Further analysis in the group of sleepy patients showed that serum 25(OH)D levels were positively correlated with average oxyhemoglobin saturation during sleep (r = 0.194, *p* = 0.043) and negatively associated with the ESS score (r = −0.285, *p* = 0.003), AHΙ (r = −0.197, *p* = 0.040) and arousal index (r = −0.256, *p* = 0.019). Conversely, among non-sleepy OSA patients, Vit D serum levels were positively associated with average oxyhemoglobin saturation (r = 0.250, *p* = 0.009) and negatively associated with time with oxyhemoglobin saturation <90% (r = −0.214, *p* = 0.028) during sleep. 

Age, sex, BMI, indices of oxygenation during sleep (average and minimum oxyhemoglobin saturation and time with oxyhemoglobin saturation <90%), total sleep time, sleep efficiency, AHI, arousal index and Vit D serum levels were included in a binary logistic regression analysis model in order to identify independent predictors of EDS. This analysis revealed that Vit D serum levels (β = −0.045, OR: 0.956, 95% CI: 0.916–0.997, *p* = 0.035), total sleep time (β = 0.011, OR: 1.011, 95% CI: 1.002–1.021, *p* = 0.016) and AHI (β = 0.022, OR: 1.022, 95% CI: 1.003–1.043, *p* = 0.026) emerged as independent predictors of EDS in patients with OSA. 

In the group of OSA patients with EDS, correlations of ESS score with other indicators were determined using a multivariate linear regression analysis. In this analysis, ESS score was set as the outcome, whereas age, sex, BMI, indices of oxygenation during sleep (average and minimum oxyhemoglobin saturation and time with oxyhemoglobin saturation <90%), total sleep time, sleep efficiency, AHI, arousal index and Vit D serum levels were set as covariates (regression equation: y = 13.255 − 0.02a − 0.254b + 0.041c + 0.204d − 0.137e + 0.008f + 0.001g − 0.034h − 0.029j + 0.033k − 0.135m). Results indicated that the ESS score was negatively associated with Vit D serum levels (β = −0.135, *p* = 0.014) and minimum oxyhemoglobin saturation during sleep (β = −0.137, *p* = 0.043). In the group of OSA patients without EDS, a similar analysis showed that the ESS score was positively associated with sleep efficiency (β = 0.138, *p* = 0.001).

## 4. Discussion

The present study reported significantly lower levels of 25(OH)D in patients with OSA and EDS compared with patients with OSA and without EDS. Additionally, Vit D serum levels, AHI and total sleep time were identified as independent predictors of EDS. Finally, 25(OH)D levels were associated with indices of hypoxia during sleep and total sleep time.

Both decreased serum 25(OH)D levels and the severity of OSA were associated with EDS in the group of patients with OSA. The relationship between EDS and hypovitaminosis D has been a subject of interest in previous studies. In a previous study, an association was shown between Vit D status and excessive daytime sleepiness in patients with sleep disorders, of which OSA was the most prevalent [17]. Interestingly, only in Black patients with Vit D deficiency (defined as <20 ng/mL) were Vit D levels correlated with sleepiness (r = 0.48, *p* < 0.05), expressed as scores in ESS ≥ 10 [17]. In addition, multiple lines of evidence indicate that patients with OSA are more prone to Vit D deficiency than those without OSA [18,19], and that treatment with CPAP could increase Vit D levels either after short-term application and in male patients with OSA [20], and after long-term application in sleepy patients and those with severe OSA [21], or more specifically in male obese patients with OSA [12]. However, the association between Vit D and OSA remains controversial. In a recent study that included 133 patients suspected of having OSA, no difference was noted between hypertensive and normotensive subjects [22]. Polysomnography was conducted following the classification of subjects into hypertensive and normotensive groups. Therefore, not all individuals in the study were diagnosed with OSA. The inclusion of individuals without OSA in the study population may explain the discrepancy in these findings. In the same study, a negative association between the calcium concentration and arousal index and a correlation between AHI and Vit D concentration was observed. These findings indeed suggest a potential relationship between Vitamin D and OSA [22].

In the present study, differences between sleepy and non-sleepy patients with OSA in terms of polysomnographic parameters were noted. Specifically, OSA patients with EDS exhibited an increased AHI, total sleep time, arousal index and sleep efficiency, and presented worse hypoxia during sleep compared with those individuals without EDS. These results confirm previous reports on the association of EDS (assessed either by subjective or objective tools [23]) with anthropometric and polysomnographic characteristics in OSA patients, including a higher BMI, longer total sleep time, increased arousal index and decreased minimum oxyhemoglobin saturation during REM and NREM sleep [24]. 

Hypoxia may serve as an underlying mechanism explaining the association between hypovitaminosis D and EDS. Previous studies have reported a clear link between impaired oxygenation during sleep and EDS in individuals with OSA, suggesting a potential causal relationship between nocturnal hypoxia and EDS [23,24]. Concurrently, hypoxia and hypoxia-related factors, such as hypoxia-inducible factor-1α subunit (HIF-1α) and vascular endothelial growth factor (VEGF), are inversely correlated to serum 25(OH)D levels [25,26]. Indeed, in our study we confirmed previous reports that patients with OSA exhibit decreased 25(OH)D levels in comparison to healthy controls, and these diminished 25(OH)D levels are correlated with average oxyhemoglobin saturation and with the percentage of time with oxyhemoglobin saturation <90% during sleep [18]. Thus, the over-expression of inflammatory factors promoted by hypoxia during sleep may mediate the relationship between low Vit D levels and increased levels of EDS in OSA patients.

Moreover, several other pathogenetic mechanisms have been proposed in order to elucidate the link between Vit D and EDS. An underlining hypothesis suggests that low Vit D serum levels could lead to EDS through mechanisms involving the upregulation of inflammatory mediators and hypnogenic cytokines such as TNF-α, IL-1, IL-6 and prostagladin-2 [27,28]. The relationship between EDS and increased AHI has been proven in some, but not in all studies [29,30,31]. Excluding AHI, other factors, including metabolic and psychological conditions, are associated with an increased risk of EDS in OSA patients [32]. EDS has been frequently reported among diabetic patients without OSA and constitutes a risk factor for severe hypoglycemia [28,33]. Recently, reduced serum Vit D levels have been associated with increased insulin resistance in patients with OSA [34]. Similarly, in patients with OSA and EDS associations have been shown between insulin resistance and glucose deregulation [35,36]. Moreover, in a median follow-up of 8.1 years, lower serum Vit D concentrations were associated with increased risk of type 2 diabetes, with daytime sleepiness being the major contributor [37]. Thus, insulin resistance may mediate the emergence of EDS in patients with OSA and Vit D insufficiency.

Notably, studies exploring the association between EDS and Vit D serum levels in conditions other than OSA have reported conflicting results. In the study of Carlander et al. [38], serum 25(OH)D concentrations were decreased in patients with narcolepsy compared with healthy controls. Patients with narcolepsy were at increased risk of Vit D deficiency compared with non-narcoleptic subjects (72.5% versus 50.9%, respectively) [38]. Conversely, another study showed similar levels of 25(OH) D between patients with narcolepsy type 1 and healthy controls [39]. In patients with narcolepsy, no significant association was found between Vit D deficiency and disease duration or severity [39]. OSA and narcolepsy frequently coexist (about 25%) and this fact may explain the puzzling results regarding the association between Vit D and narcolepsy [40].

Certainly, our study has a number of limitations. Firstly, our data were obtained from middle-aged adults and no generalization of the results could be performed in older patients with OSA. Of note, regression analysis failed to demonstrate age as an influencing factor for EDS in our participants. Secondly, data regarding skin pigmentation, clothing and dietary habits were not recorded in the current study. Nevertheless, the study was conducted in a short time interval (6 months), and included Caucasian patients, living in the same area, with relatively similar sun exposure and dietary habits. Additionally, the number of included female patients was relatively small and thus the study results should be interpreted with caution. However, at regression analysis, gender was excluded as a cofounder regarding the relationship between Vit D deficiency and EDS. It should be noted that the index of females/males was the result of an increased male referral and did not result from a female exclusion process. Finally, EDS was evaluated using the ESS and not an objective method, such as the multiple sleep latency test. However, there is evidence suggesting that ESS can be a valid tool for the evaluation of EDS [41]. Potential mechanisms of EDS in OSA are still not entirely clear [42].

## 5. Conclusions

In conclusion, our results show that both AHI and Vit D serum levels predict EDS in a group of patients with OSA. Hypoxia during sleep may play an important role in this process. A possible bi-directional relationship between OSA and hypovitaminosis D could partially explain our findings. Further research is needed in order to better elucidate the interaction between serum Vit D levels and EDS in patients with OSA.

## Figures and Tables

**Table 1:** Comparison of anthropometric characteristics between OSA patients with and without EDS.

	OSA Patients without EDS n = 108	OSA Patients with EDS n = 109	*p*
Gender (male/female)	96/12	101/8	0.337
Age (years)	55.5 (46–64)	54 (47–62)	0.839
BMI (kg/m^2^)	34.3 (30.7–37.7)	36 (32.1–38.9)	0.103
Neck circumference (cm)	44 (42–47)	45 (42–48)	0.502
Waist circumference (cm)	121 (113–129)	121 (112–130)	0.764
Hip circumference (cm)	116 (111–124)	116 (110–123)	0.937
WHR	1.03 (0.99–1.06)	1.03 (0.99–1.08)	0.406
Smoking (%)	24.1%	28.4%	0.465

BMI: body mass index; EDS: excessive daytime sleepiness; OSA: obstructive sleep apnea; WHR: waist-to-hip ratio.

**Table 2:** Comparison of sleep characteristics between OSA patients with and without EDS.

	OSA Patients without EDS n = 108	OSA Patients with EDS n = 109	*p*
TST (min)	311 (263–339)	340 (311–360)	<0.001
N1 (%)	12.3 (5.5–18.7)	7.7 (4.4–15.3)	0.055
N2 (%)	70.1 (59.2–77.9)	72.2 (61.9–85.1)	0.046
N3 (%)	7.5 (1.8–15.2)	5.2 (0–13.6)	0.074
REM (%)	7.5 (1.4–12.9)	5.4 (1.3–10.5)	0.171
AHI (events/h)	33.9 (15–62.3)	54.9 (35.2–73)	<0.001
Aver SpO_2_ (%)	92 (90–94)	91 (89–93)	0.002
Min SpO_2_ (%)	77 (69–82)	73 (63–79)	0.008
T < 90% (%)	11.8 (3.2–38.3)	30.9 (12.8–59.6)	<0.001
Arousal index	27.5 (13.5–35.7)	35.2 (19.2–49.5)	0.034
Sleep efficiency (%)	83.7 (75.7–90)	88.6 (80.2–92.7)	0.004
ESS score	7 (5–9)	14 (12–17)	<0.001

AHI: apnea-hypopnea index, Aver SpO_2_: average oxyhemoglobin saturation, EDS: excessive daytime sleepiness, ESS: Epworth sleepiness scale, Min SpO_2_: minimum oxyhemoglobin saturation, N1: sleep stage 1, N2: sleep stage 2, N3: sleep stage 3, OSA: obstructive sleep apnea, REM: rapid eye movement, TST: total sleep time, T < 90%: time with oxyhemoglobin saturation < 90%.

**Table 3:** Comparison of laboratory results between OSA patients with and without EDS.

	OSA Patients without EDS n = 108	OSA Patients with EDS n = 109	*p*
Glucose (mg/dL)	103 (92–117)	114 (94.3–128.8)	0.078
Creatinine (mg/dL)	0.9 (0.8–1)	0.9 (0.8–1)	0.316
Cholesterol (mg/dL)	201 (176.8–234)	198 (176–234.8)	0.735
Triglycerides (mg/dL)	136.5 (98–181)	167 (112.5–211.8)	0.013
LDL-C (mg/dL)	125.9 (96.3–151.5)	118.2 (102–142.3)	0.899
HDL-C (mg/dL)	47 (42–57.3)	42 (37–52)	0.007
AST (U/L)	23 (19–27)	21.5 (18–27.8)	0.390
ALT (U/L)	25 (17–33)	24.5 (20–35)	0.604
CRP (mg/dL)	0.25 (0.1–0.79)	0.40 (0.20–0.66)	0.376
25(OH)D (ng/mL)	21.1 (15.3–28.8)	17.4 (12.2–25.7)	0.005

ALT: alanine aminotransferase, AST: aspartate aminotransferase, CRP: C—reactive protein, HDL-C: high-density lipoprotein cholesterol, LDL-C: low-density lipoprotein cholesterol.

**Table 4:** Comparison of pulmonary function testing results between OSA patients with and without EDS.

	OSA Patients without EDS n = 108	OSA Patients with EDS n = 109	*p*
FEV_1_ (% predicted)	93.8 (81.9–103)	92.3 (75.9–106.8)	0.736
FVC (% predicted)	90.7 (78.1–99.3)	85.9 (73.8–97.8)	0.137
FEV_1_/FVC (%)	86.5 (82–110.3)	83 (79–95)	0.323
pO_2_ (mmHg)	79 (73–85.6)	78.5 (68.5–86)	0.344
pCO_2_ (mmHg)	41 (38.9–44)	42 (39–45)	0.327

FEV_1_: forced expiratory volume in 1st sec, FVC: forced vital capacity, pCO_2_: carbon dioxide partial pressure, pO_2_: oxygen partial pressure.

## Data Availability

Data Availability Statements are available upon request.
